# Peer Review of “Machine Learning–Based Hyperglycemia Prediction: Enhancing Risk Assessment in a Cohort of Undiagnosed Individuals”

**DOI:** 10.2196/60853

**Published:** 2024-09-11

**Authors:** Akhil Chaturvedi

**Affiliations:** 1 Headspace, San Francisco, CA, United States

**Keywords:** hyperglycemia, diabetes, machine learning, hypertension, random forest

*This is the peer-review report for “Machine Learning–Based Hyperglycemia Prediction: Enhancing Risk Assessment in a Cohort of Undiagnosed Individuals.”*

## Round 1 Review

### General Comments

Overall strong paper [1]! This was an interesting study on the use of machine learning to predict hyperglycemia in a cohort of undiagnosed individuals from Nigeria. I feel like this work is a strong contribution to the field of public health, especially within the context of noncommunicable diseases in developing countries. I also like that it is backed well with quantitative methods. The strengths of this manuscript lie in its detailed methodology and its comprehensive data analysis.

### Specific Comments

#### Major Comments

- While the study demonstrates a robust analytical approach, it would benefit from external validation with an independent dataset. This would strengthen the findings and ensure the model’s generalizability and applicability in different populations.
- The manuscript could be improved by providing more context on the selection of the machine learning algorithms used in the study. An explanation of why certain algorithms were chosen and others potentially excluded would offer clarity.

#### Minor Comments

- The manuscript occasionally uses technical jargon that might not be easily understandable to readers not familiar with machine learning. Simplifying the language or providing brief explanations will make the paper more accessible.
- The study’s potential for real-world application would be clearer with a section on future work, detailing how these algorithms could be deployed in clinical settings or used in larger-scale studies (I can see how this might be a tangential research direction, but this would still be great given the potential impact).

Based on this, my recommendation is:

A: Accept
